# Supplementary material for: Modeling glioblastoma heterogeneity as a dynamic network of cell states
Source: Mol Syst Biol. 2021 Sep 16;17(9):e10105. doi: 10.15252/msb.202010105 (PMC8444284; doi:10.15252/msb.202010105)
Supplement: Supplementary file 6 — Source Data for Figure 5 [file MSB-17-e10105-s004.zip › Figure5A_sourcedata/GSEA_3017/nosigdb_stateB.GseaPreranked.1622795021725/gsea_report_for_na_neg_1622795021725.html]

Report for na\_neg 1622795021725 [GSEA]

| GS  follow link to MSigDB | GS DETAILS | SIZE | ES | NES | NOM p-val | FDR q-val | FWER p-val | RANK AT MAX | LEADING EDGE || 1 | RICHARDS\_NOWAKOWSKI\_SCIENCE\_2017\_IPC\_NEN3\_UPREG | Details ... | 23 | -0.61 | -3.46 | 0.000 | 0.000 | 0.000 | 76 | tags=65%, list=10%, signal=70% |
| 2 | RICHARDS\_NOWAKOWSKI\_SCIENCE\_2017\_OPC\_UPREG | Details ... | 32 | -0.43 | -2.93 | 0.000 | 0.000 | 0.000 | 83 | tags=47%, list=11%, signal=50% |
| 3 | RICHARDS\_NOWAKOWSKI\_SCIENCE\_2017\_NEN\_EARLY1\_UPREG | Details ... | 36 | -0.40 | -2.78 | 0.000 | 0.000 | 0.000 | 78 | tags=44%, list=10%, signal=47% |
| 4 | LARSSON\_STATE\_6 | Details ... | 20 | -0.48 | -2.64 | 0.000 | 0.002 | 0.002 | 75 | tags=50%, list=10%, signal=54% |
| 5 | RICHARDS\_INHOUSE\_BULKRNASEQ\_2019\_DEVELOPMENTALGSC | Details ... | 86 | -0.25 | -2.60 | 0.000 | 0.001 | 0.002 | 199 | tags=51%, list=26%, signal=61% |
| 6 | RICHARDS\_NOWAKOWSKI\_SCIENCE\_2017\_IPC\_NEN1\_UPREG | Details ... | 24 | -0.43 | -2.55 | 0.000 | 0.002 | 0.003 | 63 | tags=46%, list=8%, signal=48% |
| 7 | RICHARDS\_NOWAKOWSKI\_SCIENCE\_2017\_ASTROCYTE\_UPREG | Details ... | 56 | -0.30 | -2.47 | 0.000 | 0.002 | 0.004 | 73 | tags=34%, list=10%, signal=35% |
| 8 | RICHARDS\_NOWAKOWSKI\_SCIENCE\_2017\_NIN1\_UPREG | Details ... | 15 | -0.49 | -2.29 | 0.000 | 0.004 | 0.007 | 227 | tags=80%, list=30%, signal=112% |
| 9 | RICHARDS\_NOWAKOWSKI\_SCIENCE\_2017\_IN\_CTX\_CGE1\_UPREG | Details ... | 21 | -0.41 | -2.24 | 0.000 | 0.005 | 0.010 | 416 | tags=95%, list=55%, signal=205% |
| 10 | DOROSZKO\_U3054MG\_CLUSTER\_8 | Details ... | 27 | -0.33 | -2.18 | 0.000 | 0.006 | 0.014 | 450 | tags=93%, list=59%, signal=219% |
| 11 | RICHARDS\_VERHAAK\_CANCERCELL\_2010\_PRONEURAL | Details ... | 18 | -0.41 | -2.18 | 0.000 | 0.006 | 0.014 | 72 | tags=50%, list=9%, signal=54% |
| 12 | RICHARDS\_NOWAKOWSKI\_SCIENCE\_2017\_EN\_PFC2\_UPREG | Details ... | 72 | -0.21 | -2.14 | 0.000 | 0.007 | 0.018 | 504 | tags=90%, list=66%, signal=243% |
| 13 | RICHARDS\_NOWAKOWSKI\_SCIENCE\_2017\_GLYC\_UPREG | Details ... | 40 | -0.28 | -2.13 | 0.000 | 0.007 | 0.018 | 389 | tags=83%, list=51%, signal=160% |
| 14 | DOROSZKO\_U3054MG\_CLUSTER\_10 | Details ... | 22 | -0.37 | -2.03 | 0.000 | 0.012 | 0.036 | 489 | tags=100%, list=64%, signal=272% |
| 15 | RICHARDS\_CAHOY\_JNEUROSCI\_2015\_ASTRO\_IN\_VIVO | Details ... | 85 | -0.19 | -1.98 | 0.000 | 0.015 | 0.048 | 616 | tags=99%, list=81%, signal=463% |
| 16 | RICHARDS\_NOWAKOWSKI\_SCIENCE\_2017\_VRG\_UPREG | Details ... | 62 | -0.23 | -1.98 | 0.000 | 0.014 | 0.048 | 81 | tags=27%, list=11%, signal=28% |
| 17 | RICHARDS\_ZHONG\_NATURE\_2018\_ASTROCYTES\_UPREG | Details ... | 37 | -0.26 | -1.97 | 0.000 | 0.014 | 0.049 | 73 | tags=30%, list=10%, signal=31% |
| 18 | NB\_KI\_CLUSTER2 | Details ... | 16 | -0.42 | -1.96 | 0.016 | 0.013 | 0.049 | 336 | tags=88%, list=44%, signal=154% |
| 19 | NB\_KI\_CLUSTER6 | Details ... | 19 | -0.37 | -1.95 | 0.005 | 0.013 | 0.051 | 414 | tags=95%, list=54%, signal=203% |
| 20 | NB\_KI\_CLUSTER7 | Details ... | 20 | -0.35 | -1.87 | 0.010 | 0.019 | 0.078 | 450 | tags=95%, list=59%, signal=227% |
| 21 | RICHARDS\_NOWAKOWSKI\_SCIENCE\_2017\_NIN2\_UPREG |  | 26 | -0.30 | -1.83 | 0.006 | 0.022 | 0.096 | 208 | tags=62%, list=27%, signal=82% |
| 22 | RICHARDS\_NOWAKOWSKI\_SCIENCE\_2017\_TRG\_UPREG |  | 122 | -0.16 | -1.79 | 0.000 | 0.028 | 0.125 | 78 | tags=22%, list=10%, signal=21% |
| 23 | RICHARDS\_NOWAKOWSKI\_SCIENCE\_2017\_EN\_V1\_3\_UPREG |  | 65 | -0.19 | -1.78 | 0.018 | 0.028 | 0.131 | 504 | tags=89%, list=66%, signal=242% |
| 24 | RICHARDS\_NOWAKOWSKI\_SCIENCE\_2017\_U4\_UPREG |  | 93 | -0.16 | -1.76 | 0.000 | 0.030 | 0.143 | 546 | tags=91%, list=72%, signal=285% |
| 25 | NB\_KI\_CLUSTER4 |  | 15 | -0.37 | -1.71 | 0.038 | 0.040 | 0.185 | 75 | tags=47%, list=10%, signal=51% |
| 26 | OLSEN\_ADRENERGIC |  | 16 | -0.36 | -1.68 | 0.040 | 0.045 | 0.216 | 75 | tags=44%, list=10%, signal=48% |
| 27 | RICHARDS\_NOWAKOWSKI\_SCIENCE\_2017\_IN\_CTX\_MGE2\_UPREG |  | 17 | -0.33 | -1.68 | 0.024 | 0.044 | 0.217 | 310 | tags=76%, list=41%, signal=126% |
| 28 | NB\_KI\_CLUSTER23 |  | 22 | -0.31 | -1.68 | 0.031 | 0.042 | 0.218 | 467 | tags=95%, list=61%, signal=240% |
| 29 | RICHARDS\_NOWAKOWSKI\_SCIENCE\_2017\_NEN\_LATE\_UPREG |  | 17 | -0.34 | -1.66 | 0.014 | 0.046 | 0.240 | 135 | tags=47%, list=18%, signal=56% |
| 30 | RICHARDS\_NOWAKOWSKI\_SCIENCE\_2017\_IN\_CTX\_MGE1\_UPREG |  | 23 | -0.29 | -1.64 | 0.033 | 0.051 | 0.271 | 194 | tags=57%, list=26%, signal=74% |
| 31 | RICHARDS\_NOWAKOWSKI\_SCIENCE\_2017\_IPC\_NEN1\_UPREG\_1 |  | 18 | -0.32 | -1.61 | 0.033 | 0.056 | 0.303 | 49 | tags=33%, list=6%, signal=35% |
| 32 | RICHARDS\_CAHOY\_JNEUROSCI\_2009\_NEURON |  | 72 | -0.17 | -1.60 | 0.000 | 0.056 | 0.310 | 549 | tags=92%, list=72%, signal=299% |
| 33 | RICHARDS\_NOWAKOWSKI\_SCIENCE\_2017\_EN\_V1\_2\_UPREG |  | 54 | -0.17 | -1.49 | 0.056 | 0.099 | 0.481 | 150 | tags=39%, list=20%, signal=45% |
| 34 | RICHARDS\_NOWAKOWSKI\_SCIENCE\_2017\_EN\_PFC3\_UPREG |  | 29 | -0.23 | -1.46 | 0.087 | 0.111 | 0.534 | 78 | tags=31%, list=10%, signal=33% |
| 35 | RICHARDS\_NOWAKOWSKI\_SCIENCE\_2017\_NEN\_EARLY2\_UPREG |  | 26 | -0.24 | -1.44 | 0.058 | 0.119 | 0.566 | 24 | tags=19%, list=3%, signal=19% |
| 36 | RICHARDS\_CAHOY\_JNEUROSCI\_2013\_OL\_MYEL |  | 50 | -0.17 | -1.34 | 0.146 | 0.182 | 0.740 | 455 | tags=82%, list=60%, signal=191% |
| 37 | RICHARDS\_NOWAKOWSKI\_SCIENCE\_2017\_EN\_V1\_1\_UPREG |  | 59 | -0.15 | -1.30 | 0.141 | 0.210 | 0.788 | 246 | tags=53%, list=32%, signal=72% |
| 38 | RICHARDS\_NOWAKOWSKI\_SCIENCE\_2017\_NIN5\_UPREG |  | 17 | -0.26 | -1.30 | 0.148 | 0.212 | 0.804 | 75 | tags=35%, list=10%, signal=38% |
| 39 | RICHARDS\_NOWAKOWSKI\_SCIENCE\_2017\_ORG\_UPREG |  | 129 | -0.10 | -1.23 | 0.050 | 0.272 | 0.880 | 578 | tags=90%, list=76%, signal=312% |
| 40 | DOROSZKO\_U3180MG\_CLUSTER\_2 |  | 28 | -0.18 | -1.19 | 0.209 | 0.311 | 0.923 | 627 | tags=100%, list=83%, signal=550% |
| 41 | NB\_KI\_CLUSTER3 |  | 20 | -0.22 | -1.19 | 0.250 | 0.304 | 0.923 | 600 | tags=100%, list=79%, signal=463% |
| 42 | RICHARDS\_NOWAKOWSKI\_SCIENCE\_2017\_ENDOTHELIAL\_UPREG |  | 105 | -0.10 | -1.15 | 0.190 | 0.333 | 0.948 | 69 | tags=17%, list=9%, signal=16% |
| 43 | NB\_KI\_CLUSTER14 |  | 16 | -0.24 | -1.13 | 0.293 | 0.362 | 0.958 | 149 | tags=50%, list=20%, signal=61% |
| 44 | RICHARDS\_NOWAKOWSKI\_SCIENCE\_2017\_EN\_PFC1\_UPREG |  | 58 | -0.12 | -1.11 | 0.286 | 0.381 | 0.967 | 453 | tags=79%, list=60%, signal=181% |
| 45 | RICHARDS\_CAHOY\_JNEUROSCI\_2008\_OLIGODENDROCYTE |  | 74 | -0.09 | -1.01 | 0.526 | 0.525 | 0.992 | 55 | tags=14%, list=7%, signal=13% |
| 46 | DOROSZKO\_U3054MG\_CLUSTER\_2 |  | 34 | -0.15 | -1.00 | 0.438 | 0.533 | 0.995 | 290 | tags=59%, list=38%, signal=91% |
| 47 | RICHARDS\_NOWAKOWSKI\_SCIENCE\_2017\_MURAL\_UPREG |  | 60 | -0.11 | -0.96 | 0.543 | 0.587 | 0.996 | 563 | tags=90%, list=74%, signal=320% |
| 48 | RICHARDS\_NOWAKOWSKI\_SCIENCE\_2017\_U1\_UPREG |  | 109 | -0.08 | -0.95 | 0.538 | 0.593 | 0.996 | 694 | tags=99%, list=91%, signal=977% |
| 49 | DOROSZKO\_U3054MG\_CLUSTER\_9 |  | 23 | -0.16 | -0.88 | 0.626 | 0.694 | 1.000 | 130 | tags=35%, list=17%, signal=41% |
| 50 | NB\_KI\_CLUSTER8 |  | 22 | -0.16 | -0.88 | 0.652 | 0.684 | 1.000 | 424 | tags=77%, list=56%, signal=170% |
| 51 | RICHARDS\_NOWAKOWSKI\_SCIENCE\_2017\_MGE\_RG1\_UPREG |  | 49 | -0.11 | -0.88 | 0.615 | 0.675 | 1.000 | 616 | tags=96%, list=81%, signal=474% |
| 52 | RICHARDS\_NOWAKOWSKI\_SCIENCE\_2017\_IN\_CTX\_CGE2\_UPREG |  | 23 | -0.14 | -0.82 | 0.745 | 0.757 | 1.000 | 591 | tags=96%, list=78%, signal=417% |
| 53 | RICHARDS\_CAHOY\_JNEUROSCI\_2011\_ASTRO\_MATURE |  | 34 | -0.12 | -0.79 | 0.812 | 0.789 | 1.000 | 303 | tags=62%, list=40%, signal=98% |
| 54 | VANGRONINGEN\_MES |  | 48 | -0.09 | -0.73 | 0.841 | 0.855 | 1.000 | 694 | tags=100%, list=91%, signal=1079% |
| 55 | RICHARDS\_NOWAKOWSKI\_SCIENCE\_2017\_MICROGLIA\_UPREG |  | 45 | -0.08 | -0.62 | 0.988 | 0.944 | 1.000 | 262 | tags=49%, list=34%, signal=70% |
Table: Gene sets enriched in phenotype **na **[plain text format]****
